# Supplementary material for: Real-time tracking and prediction of COVID-19 infection using digital proxies of population mobility and mixing
Source: Nat Commun. 2021 Mar 8;12:1501. doi: 10.1038/s41467-021-21776-2 (PMC7940469; doi:10.1038/s41467-021-21776-2)
Supplement: Supplementary file 1 — Supplementary Information [file 41467_2021_21776_MOESM1_ESM.pdf]

Supplementary Information

Real-time tracking and prediction of COVID-19 infection using digital proxies of population mobility and mixing

Kathy Leung[1,2*], Joseph T Wu[1,2*], Gabriel M Leung[1,2]

[1]WHO Collaborating Centre for Infectious Disease Epidemiology and Control, School of Public Health, LKS Faculty of Medicine, The University of Hong Kong, Hong Kong SAR, China

[2]Laboratory of Data Discovery for Health (D^{2}4H), Hong Kong Science Park, Hong Kong SAR, China

[*]Contributed equally.

Corresponding author: Joseph T. Wu, School of Public Health, LKS Faculty of Medicine, The University of Hong Kong, 2/F North Wing, Patrick Manson Building, 7 Sassoon Road, Pok Fu Lam, Hong Kong. Tel: + 852 3917 6709; Email: joewu@hku.hk

Supplementary Table 1. Parameters fitted in the SIR model

Parameters	Description
γ_a , $a = 1, \ldots, 4$	The scaling factors for translating the age-specific digital proxies into the contact matrix
M	The number of local infections on 22 January when the simulation was started
p_{report}	The proportion of infections ascertained by the Centre for Health Protection

Supplementary Table 2. Contact matrix of reported contacts for participants in Hong Kong in 2015-2016, consisting of the average number of contacts per day recorded by the survey participant, stratified by the age group of the contact [1]

		Age of contact			
		**0-11**	**12-18**	**19-64**	**65+**
**Age of participant**	**0-11**	3.48	0.50	4.71	0.36
	**12-18**	0.20	3.58	4.01	0.16
	**19-64**	0.38	0.61	6.13	0.42
	**65+**	0.12	0.07	3.99	0.98

Supplementary Table 3. Household contact matrix of reported household contacts for participants in Hong Kong in 2015-2016, consisting of the average number of household contacts per day recorded by the survey participant, stratified by the age group of the contact [1]

		Age of contact			
		**0-11**	**12-18**	**19-64**	**65+**
**Age of participant**	**0-11**	0.35	0.15	2.20	0.23
	**12-18**	0.12	0.23	1.41	0.10
	**19-64**	0.24	0.17	1.37	0.22
	**65+**	0.08	0.04	0.95	0.28

Supplementary Table 4. Estimated contact matrix by age in Hong Kong from the base case model, consisting of the average number of contacts made per day by an individual (that might generate infections if he/she is infectious) *, without assumptions about contact patterns inferred from survey data

		**Age of contact**			
		**0-11**	**12-18**	**19-64**	**65+**
**Age of participant**	**0-11**	0.46 (0.06-0.70)	0.42 (0.17-0.54)	0.11 (0.04-0.14)	0.10 (0.02-0.24)
	**12-18**	0.38 (0.16-0.48)	0.40 (0.17-0.55)	0.10 (0.06-0.12)	0.11 (0.02-0.16)
	**19-64**	0.88 (0.33-1.12)	0.89 (0.53-1.06)	0.22 (0.17-0.28)	0.24 (0.04-0.37)
	**65+**	0.16 (0.03-0.39)	0.21 (0.03-0.29)	0.05 (0.01-0.08)	0.06 (0.01-0.15)

* Estimated in the base case

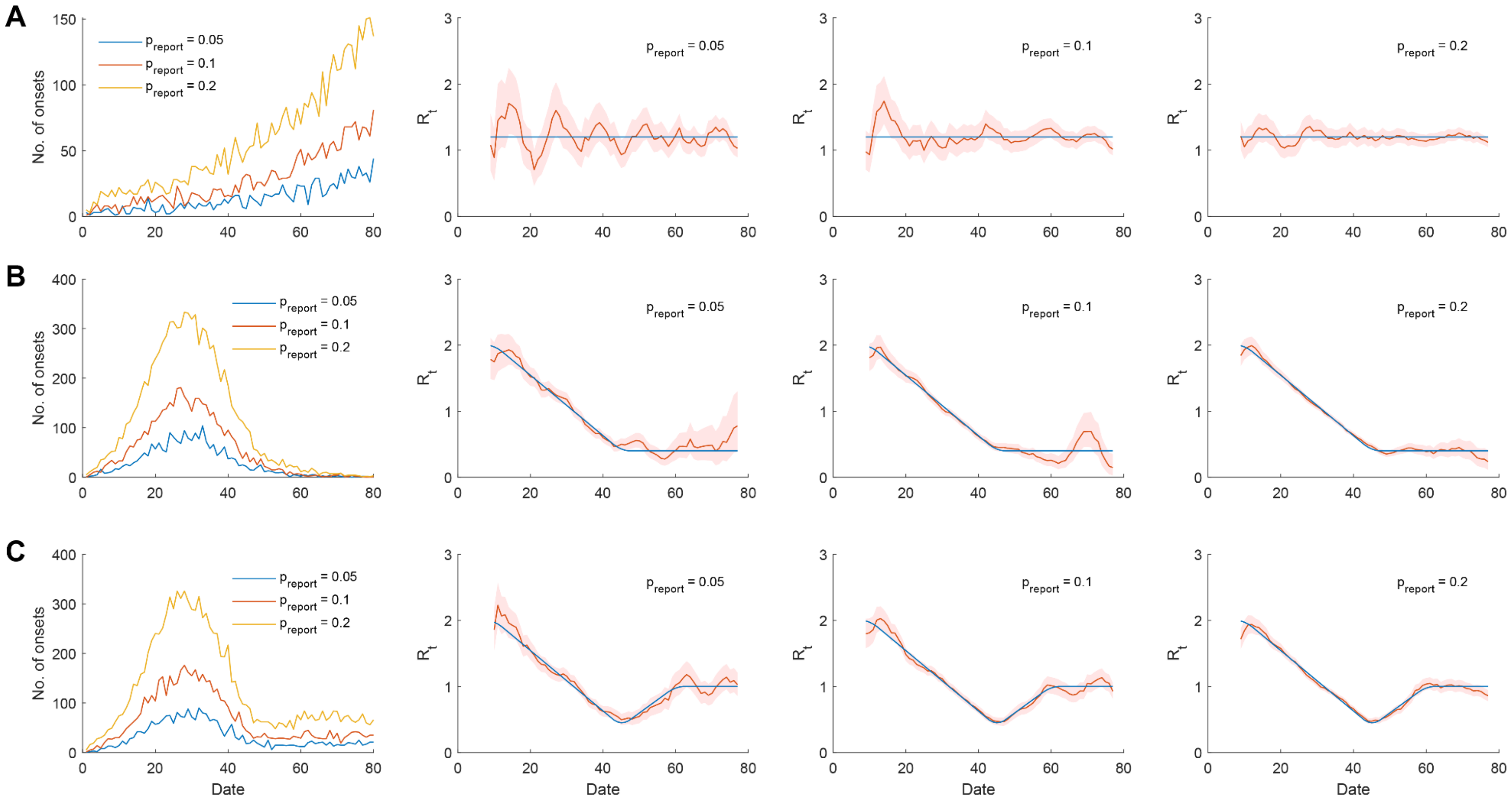

Supplementary Figure 1. Validating the accuracy of R_t estimation with deconvoluted epidemic curves. The epidemic was simulated using an SIR model seeded with 200 infectious individuals. The daily number of reported symptom onsets were simulated assuming the reporting rate p_{report} was 0.05, 0.1 and 0.2, respectively. R_t was estimated using methods from Thompson et al from deconvoluted epidemic curves of reported symptom onsets on weekly sliding windows. Blue lines indicate the true R_t by dates of infection. Red lines and shades indicate the posterior mean and 95% credible intervals of R_t estimates. (A-C) Three simulated epidemic scenarios with different true R_t over time using an SIR model seeded with 200 infectious individuals.

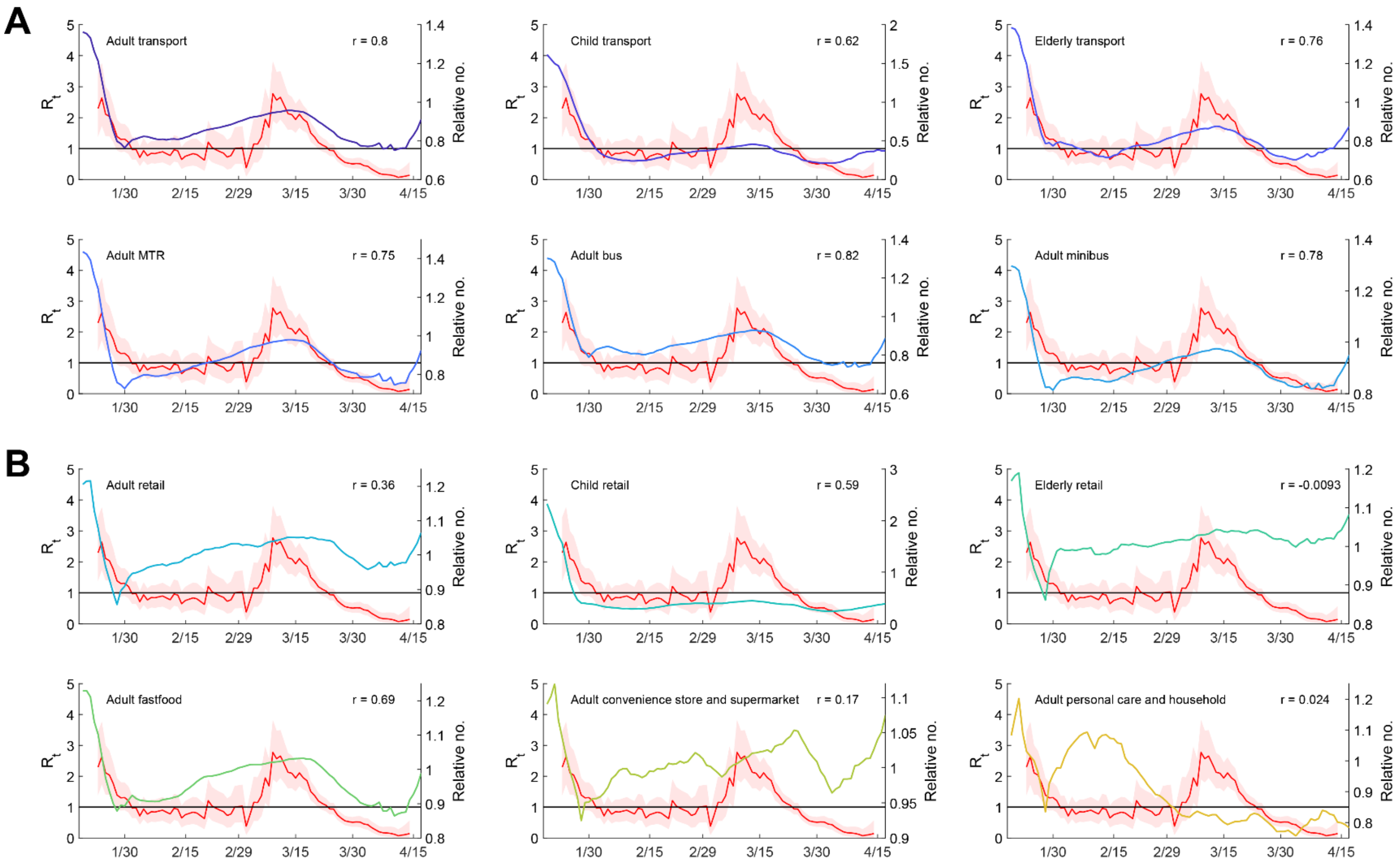

Supplementary Figure 2. The correlation between transmissibility of COVID-19 and mobility and activity inferred from different categories of Octopus data in Hong Kong. Local R_t by dates of infections were estimated on sliding weekly windows using methods from Thompson et al. R_t was estimated from local cases only (i.e., including categories of local case, possibly local case, epidemiologically linked with local case and epidemiologically linked with possibly local case). Red lines and shades indicate the posterior mean and 95% credible intervals of empirical R_t estimates. (A) Correlation with Octopus transport data (7-day moving average). (B) Correlation with Octopus retail data (7-day moving average).

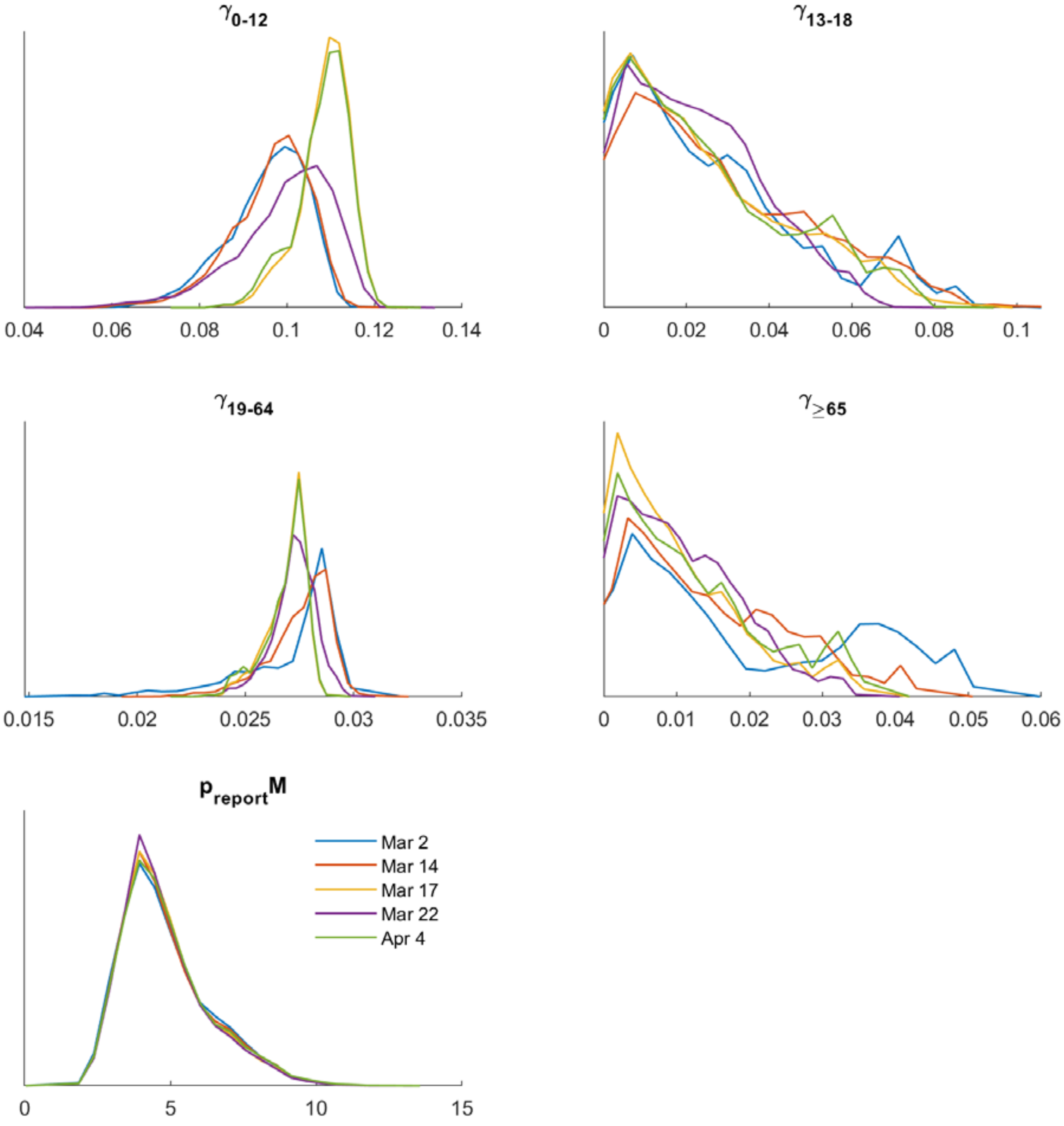

Supplementary Figure 3. The posterior distribution of model parameters on 2 March, 14 March, 17 March, 22 March and 4 April. We assumed that the generation time distribution was gamma with mean 5.2 days and coefficient of variation 0.33 in the base case.

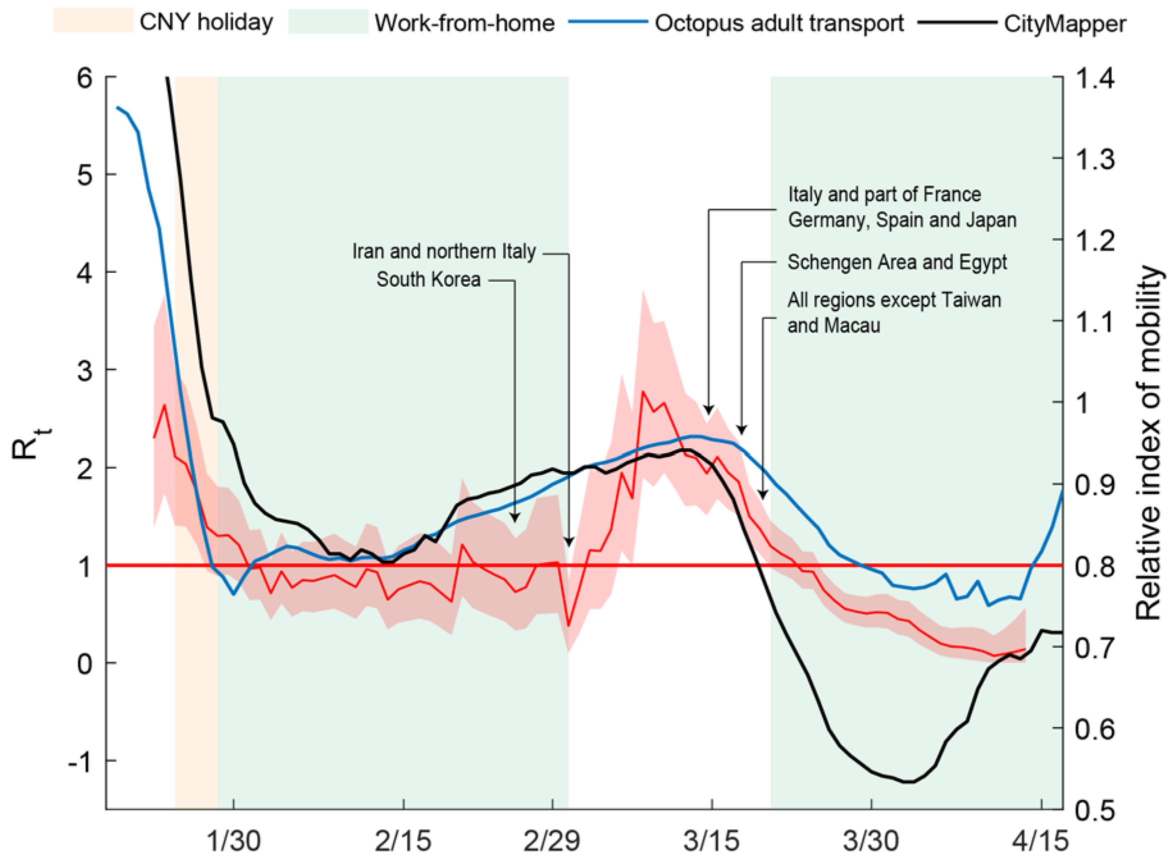

Supplementary Figure 4. The correlation between local R_t and the mobility inferred from Octopus data and CityMapper mobility index in Hong Kong. Red lines and shades indicate the posterior mean and 95% credible intervals of empirical R_t estimates. The light shades show the Chinese New Year holidays and the time period when "work-from-home" arrangements were implemented for civil servants. Mandatory 14-day quarantine were implemented for travelers from South Korea since 25 Feb, from Iran and northern Italy since 1 Mar, from Italy and affected area in France, Germany, Spain and Japan since 14 Mar, from Schengen area and Egypt since 17 Mar, from all regions and countries except Taiwan and Macau since 19 Mar, and from all regions and countries since 25 Mar. R_t was more closely correlated with mobility measured by Octopus adult transport data (r = 0.80) than CityMapper's mobility index (r = 0.67).

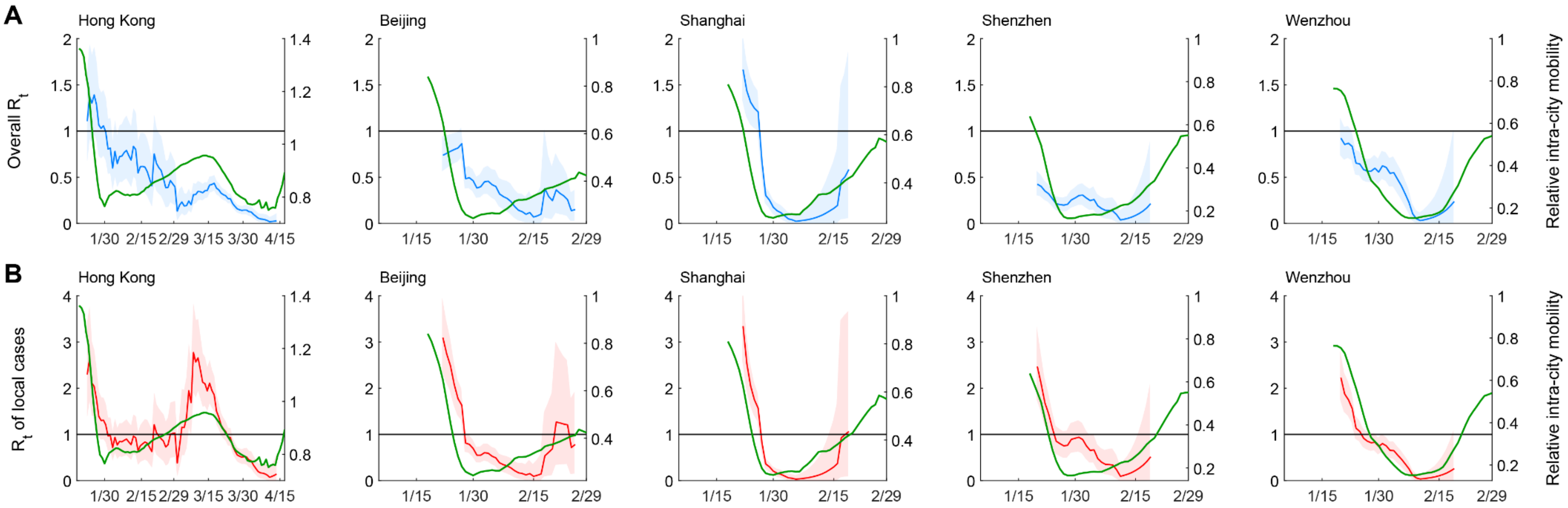

Supplementary Figure 5. Correlation between R_t of local cases and intra-city mobility indices in Hong Kong, Beijing, Shanghai, Shenzhen and Wenzhou. (A) Correlation between overall R_t and intra-city mobility indices. Octopus adult transport data were used for Hong Kong and Baidu's intra-city mobility indices were used for Beijing, Shanghai, Shenzhen and Wenzhou. Blue lines and shades show the posterior mean and 95% CrI of the overall R_t estimates. Green lines show the intra-city mobility indices. (B) Correlation between local R_t and intra-city mobility indices. Red lines and shades showed the posterior mean and 95% CrI of the local R_t estimates. Local R_t in Hong Kong were the same as Figure 1. Local R_t of the first wave in Beijing, Shanghai, Shenzhen and Wenzhou were estimated assuming that imported cases did not generate any secondary cases. R_t could only be estimated up to mid-February in most cities and provinces outside Hubei because there were very few confirmed cases reported after late-February. The Pearson's correlation coefficients between the local R_t estimates and the mobility indices were 0.72, 0.85, 0.71, 0.94 for Beijing, Shanghai, Shenzhen, and Wenzhou, respectively.

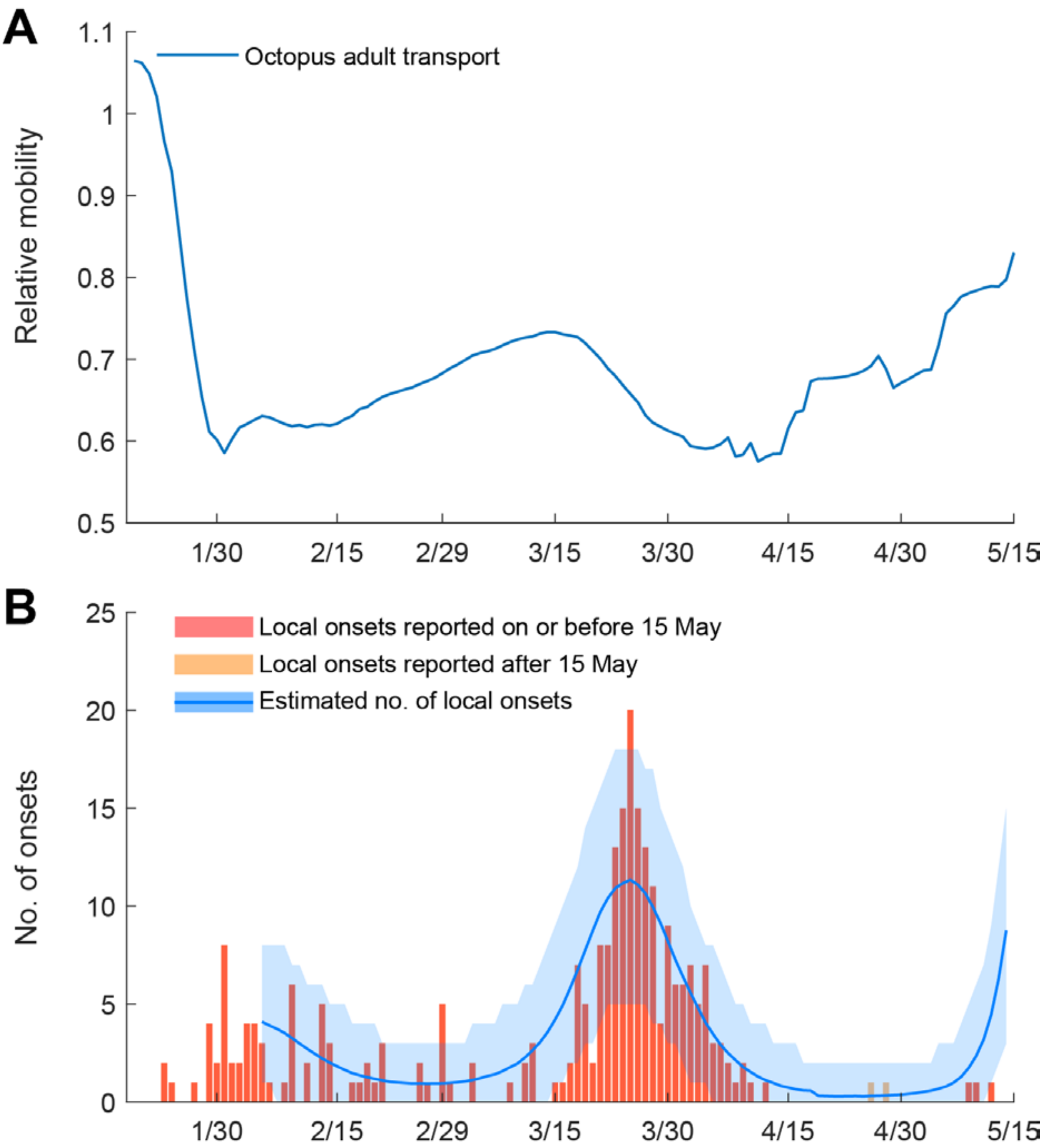

Supplementary Figure 6. The estimated epidemic curve of local reported onsets before 15 May. We fit the model to local case data between 22 January and 15 May. We estimated that the ascertainment proportion (i.e., infection-reporting probability) were 23% (13%-47%). (A) The Octopus transport data of adults between 22 January and 15 May. (B) The estimated epidemic curve of local reported onsets between 1 February and 15 May. Blue lines and shades indicate the nowcasted local epidemic curve (posterior mean and 95% credible intervals).

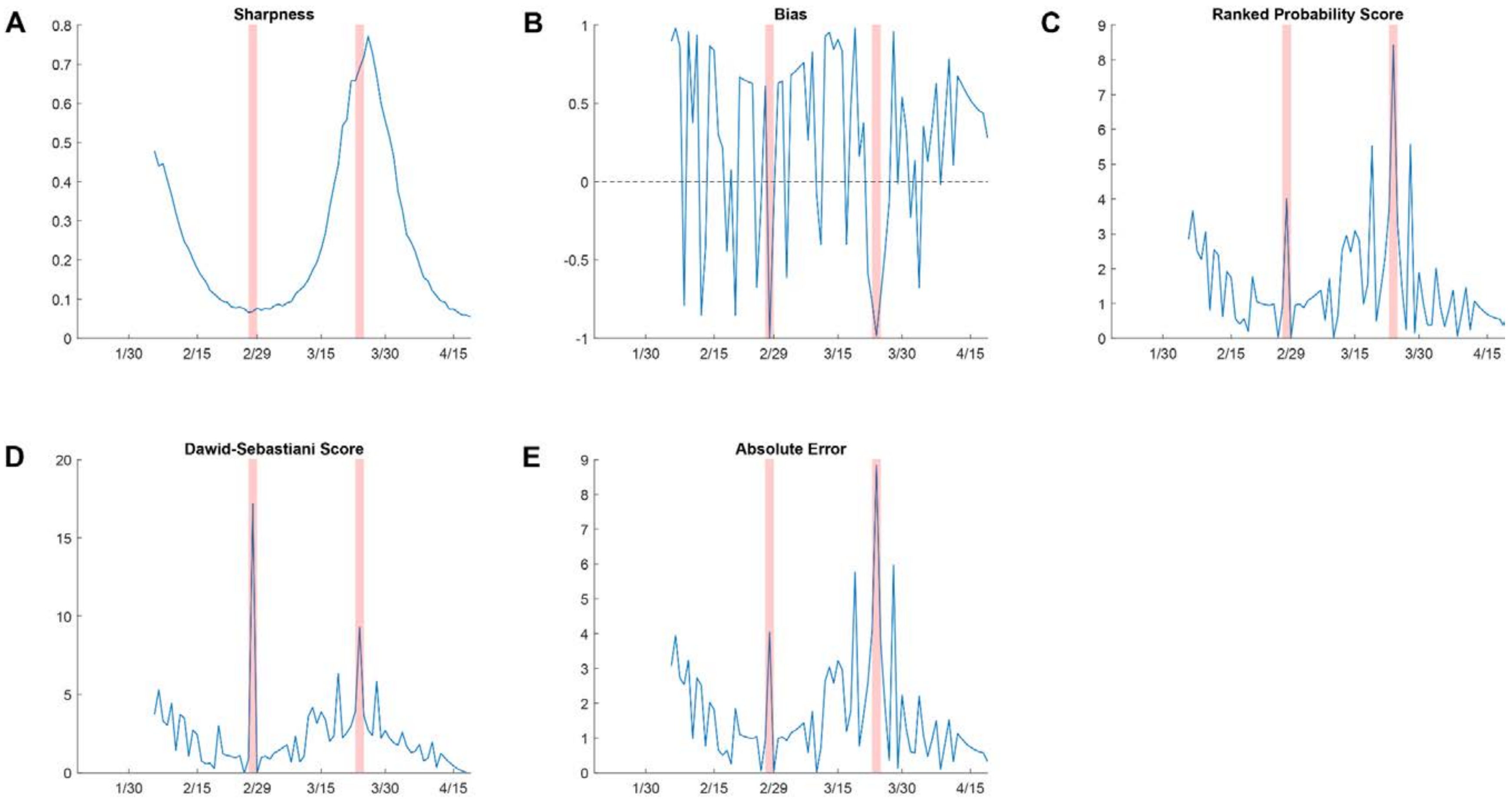

Supplementary Figure 7. Forecasting metrics and scores of the forecast performed 9 days ahead of the time point shown on the x-axis [2]. (A) Sharpness (values closer to 0 indicate sharper models); (B) Bias (values closer to 0 indicate less bias); (C) Ranked probability score (values closer to 0 indicate better forecast); (D) Dawid-Sebastiani score (values closer to 0 indicate better forecast); (E) Absolute error (values closer to 0 indicate better forecast). The light red shades indicated that the forecast model did not perform well on 29 February and 22-23 March due to the occurrence of large clusters or superspreading events.

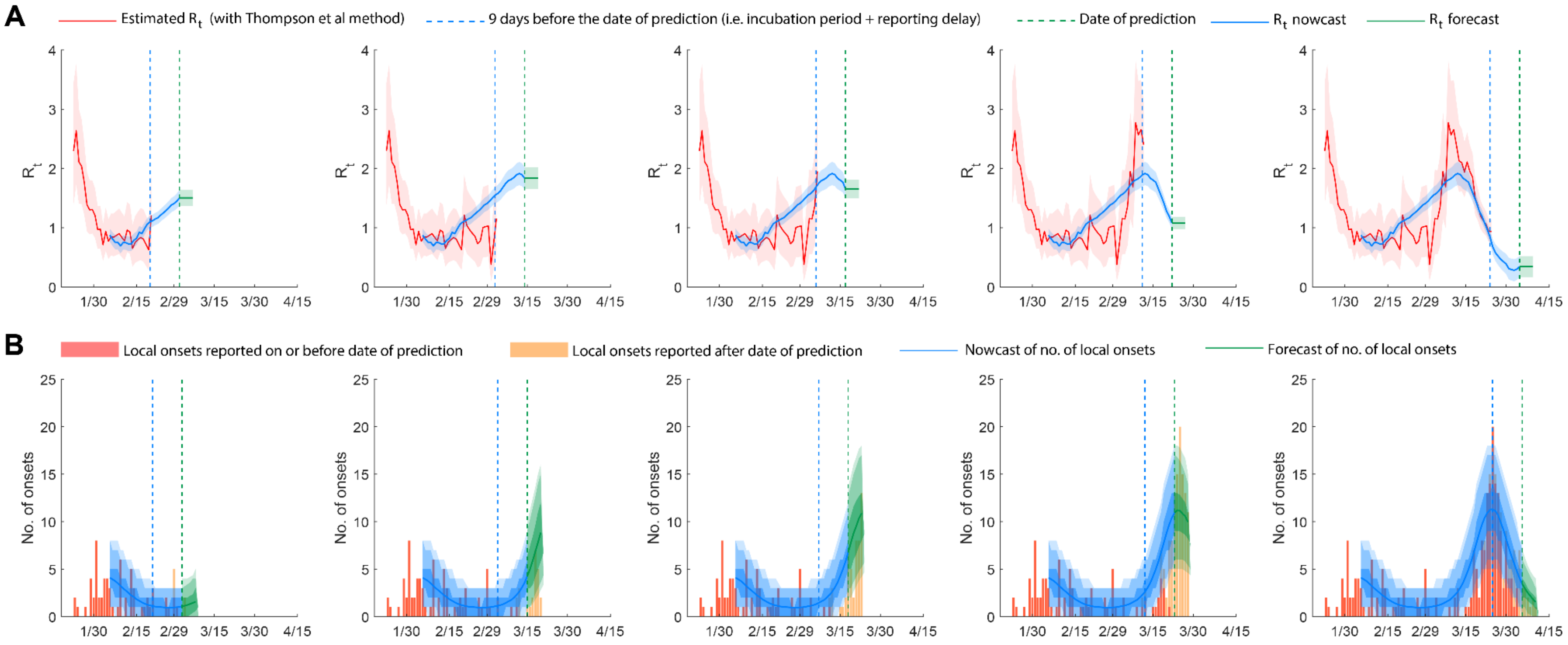

Supplementary Figure 8. Retrospective nowcast and forecast of the COVID-19 epidemic in Hong Kong. The green dash lines indicate the dates on which retrospective nowcast, and 6-day forecast were made (2 March, 14 March, 17 March, 22 March and 4 April). The blue dash lines indicate the latest empirical Rt estimates obtainable from the epidemic curve on those dates. (A) Comparison between empirical R_t estimates and R_t from the fitted SIR model. The red line and shades indicate the empirical R_t estimates. Blue lines and shades indicate the nowcasted R_t. Green lines and shades correspond to the assumption that population mixing (and hence R_t) would remain at status quo for the next 6 days. (B) Nowcast and forecast of local epidemic curves by dates of symptom onset. Red bars showed the number of local onsets reported on or before the date of prediction. Orange bars showed the number of local onsets reported after the date of prediction. Blue lines and shades (i.e., showing 50%, 90% and 95% CrIs of posterior distribution from lighter to darker shades) indicate the nowcasted local epidemic curve. Green lines and shades (i.e., showing 50%, 90% and 95% CrIs of posterior distribution from lighter to darker shades) indicate the forecasted local epidemic curve.

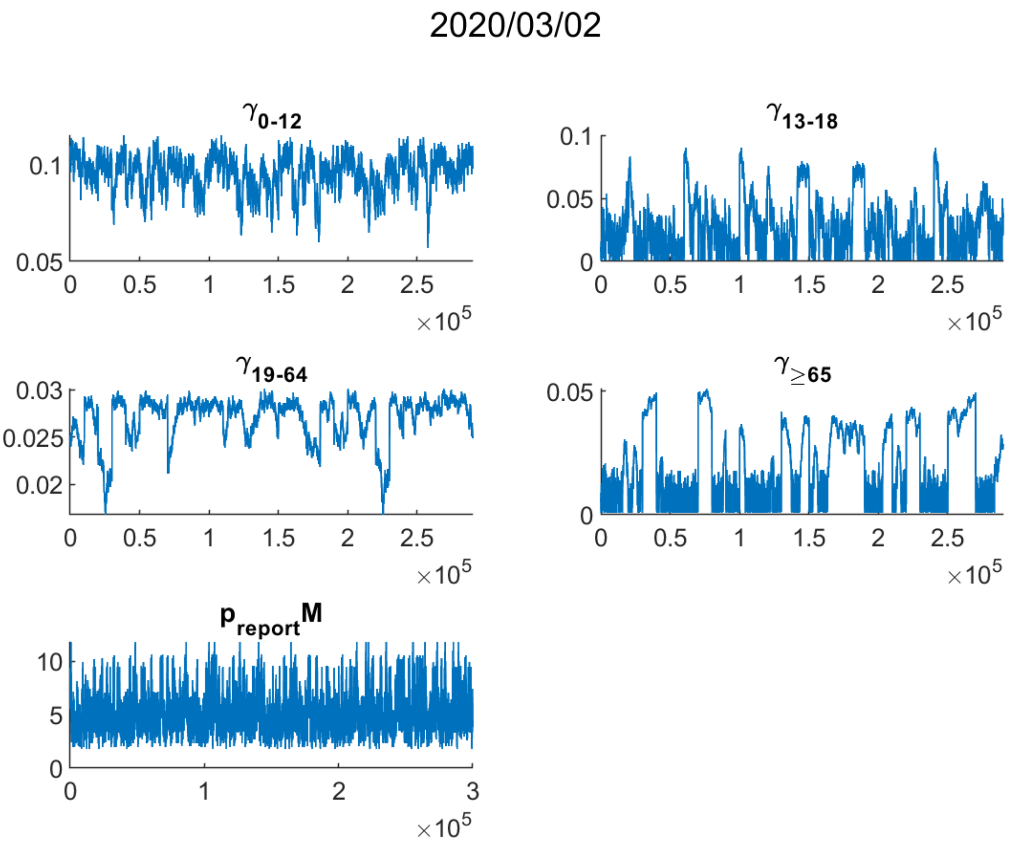
2020/03/02
γ0-12
γ13-18
γ19-64
γ≥65
preportM
×10^5

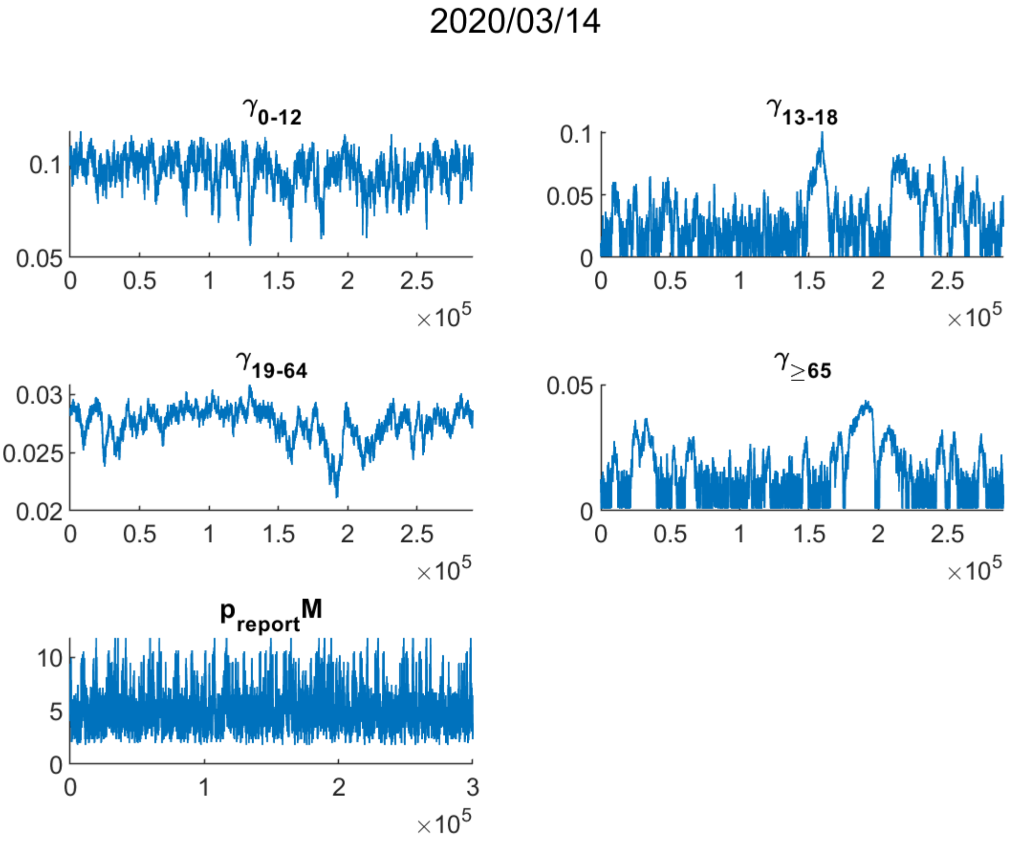
2020/03/14
γ0-12
γ13-18
γ19-64
γ≥65
preportM
×10^5

2020/03/17

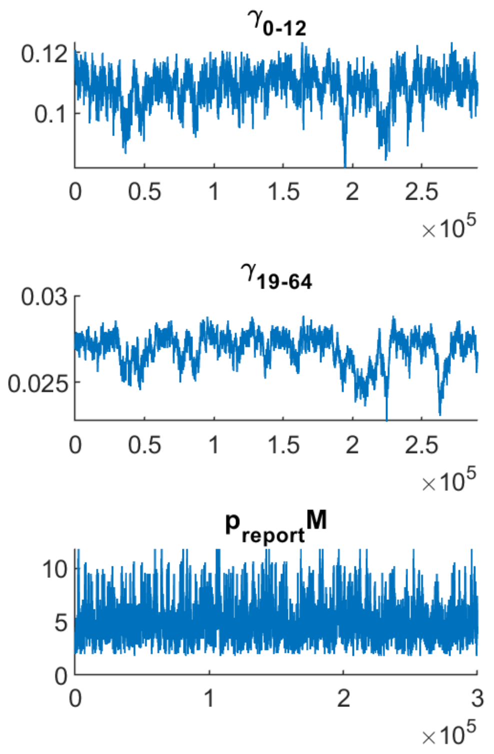

2020/03/22

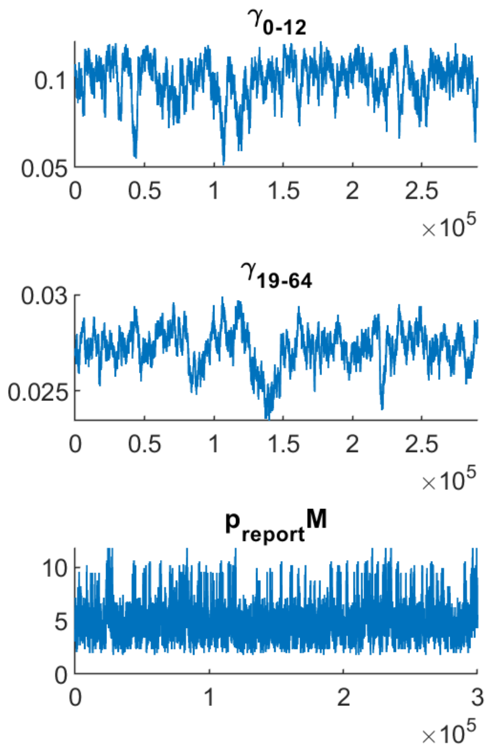

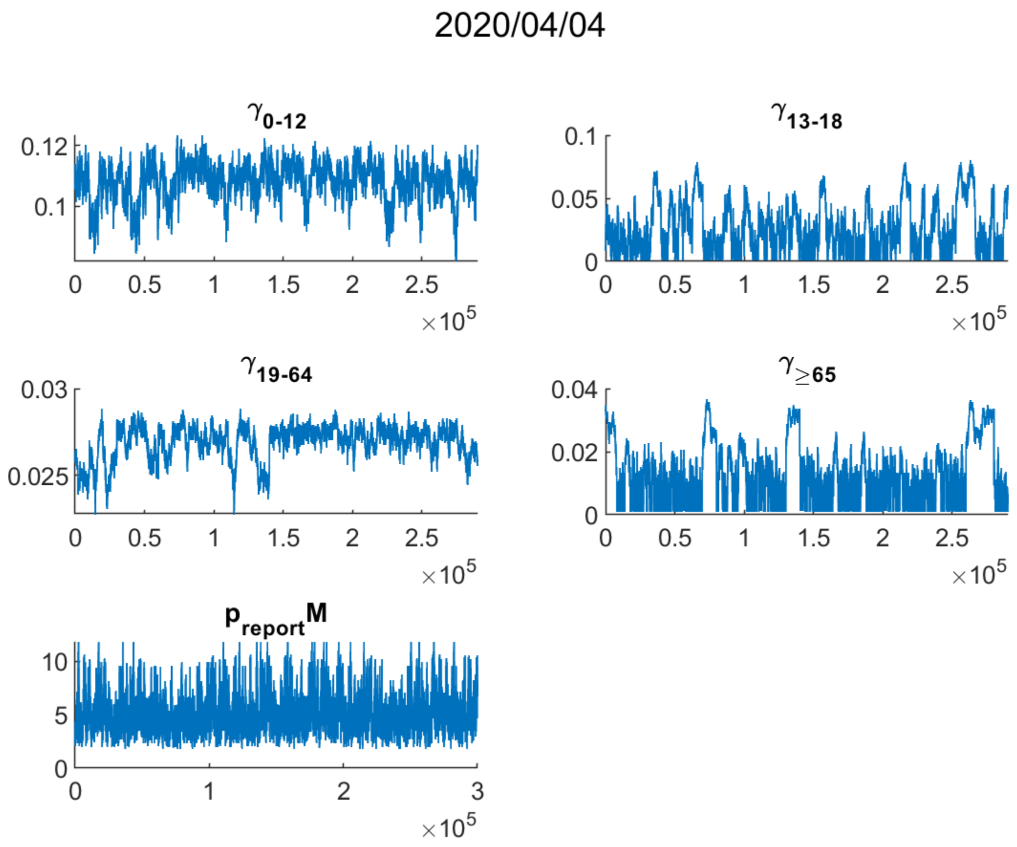

Supplementary Figure 9. The MCMC traceplots of model parameters on 2 March, 14 March, 17 March, 22 March and 4 April. We assumed that the generation time distribution was gamma with mean 5.2 days and coefficient of variation 0.33 in the base case.

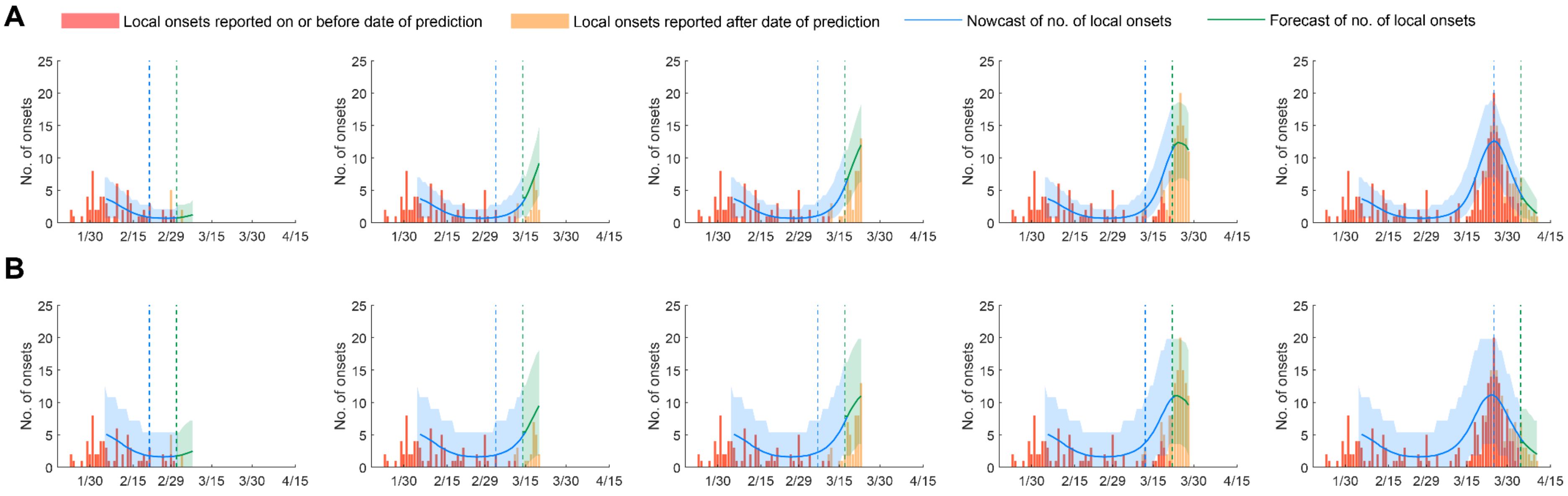

Supplementary Figure 10. Retrospective nowcast and forecast of the COVID-19 epidemic in Hong Kong. Nowcast and forecast of local epidemic curves by dates of symptom onset. Red bars showed the number of local onsets reported on or before the date of prediction. Orange bars showed the number of local onsets reported after the date of prediction. Blue lines and shades indicate the nowcasted local epidemic curve (posterior mean and 95% credible intervals). Green lines and shades indicate the forecasted local epidemic curve (posterior mean and 95% credible intervals). (A) Assuming that the generation time distribution was gamma with mean 4.2 days and coefficient of variation 0.33. (B) Assuming that the generation time distribution was gamma with mean 6.2 days and coefficient of variation 0.33.

Supplementary References

1 Leung, K., Jit, M., Lau, E. H. & Wu, J. T. Social contact patterns relevant to the spread of respiratory infectious diseases in Hong Kong. *Scientific reports* **7**, 1-12 (2017).

2 Funk, S. *et al.* Assessing the performance of real-time epidemic forecasts: A case study of Ebola in the Western Area region of Sierra Leone, 2014-15. *PLOS Computational Biology* **15**, e1006785, doi:10.1371/journal.pcbi.1006785 (2019).
