# Supplementary material for: Real-time tracking and prediction of COVID-19 infection using digital proxies of population mobility and mixing
Source: Nat Commun. 2021 Mar 8;12:1501. doi: 10.1038/s41467-021-21776-2 (PMC7940469; doi:10.1038/s41467-021-21776-2)
Supplement: Supplementary file 3 — Reporting Summary [file 41467_2021_21776_MOESM3_ESM.pdf]

nature research

Corresponding author(s): Joseph T. Wu

Last updated by author(s): Feb 1, 2021

Reporting Summary

Nature Research wishes to improve the reproducibility of the work that we publish. This form provides structure for consistency and transparency in reporting. For further information on Nature Research policies, see our Editorial Policies and the Editorial Policy Checklist.

Statistics

For all statistical analyses, confirm that the following items are present in the figure legend, table legend, main text, or Methods section.

n/a	Confirmed	
☐	☒	The exact sample size (*n*) for each experimental group/condition, given as a discrete number and unit of measurement
☒	☐	A statement on whether measurements were taken from distinct samples or whether the same sample was measured repeatedly
☐	☒	The statistical test(s) used AND whether they are one- or two-sided<br>*Only common tests should be described solely by name; describe more complex techniques in the Methods section.*
☐	☒	A description of all covariates tested
☐	☒	A description of any assumptions or corrections, such as tests of normality and adjustment for multiple comparisons
☐	☒	A full description of the statistical parameters including central tendency (e.g. means) or other basic estimates (e.g. regression coefficient) AND variation (e.g. standard deviation) or associated estimates of uncertainty (e.g. confidence intervals)
☐	☒	For null hypothesis testing, the test statistic (e.g. *F*, *t*, *r*) with confidence intervals, effect sizes, degrees of freedom and *P* value noted<br>*Give P values as exact values whenever suitable.*
☐	☒	For Bayesian analysis, information on the choice of priors and Markov chain Monte Carlo settings
☒	☐	For hierarchical and complex designs, identification of the appropriate level for tests and full reporting of outcomes
☐	☒	Estimates of effect sizes (e.g. Cohen's *d*, Pearson's *r*), indicating how they were calculated

Our web collection on statistics for biologists contains articles on many of the points above.

Software and code

Policy information about availability of computer code

Data collection	Data and codes used in the manuscript are available in: https://github.com/kathyleung/Octopus_mobility_model.
Data analysis	R 4.0.0 and MATLAB 2020a are used in the data analysis. R package "EpiEstim" 2.2-3 is used in the data analysis. R and MATLAB codes are provided in the supplementary information and in the GitHub repository https://github.com/kathyleung/Octopus_mobility_model.

For manuscripts utilizing custom algorithms or software that are central to the research but not yet described in published literature, software must be made available to editors and reviewers. We strongly encourage code deposition in a community repository (e.g. GitHub). See the Nature Research guidelines for submitting code & software for further information.

Data

Policy information about availability of data

All manuscripts must include a data availability statement. This statement should provide the following information, where applicable:

- Accession codes, unique identifiers, or web links for publicly available datasets
- A list of figures that have associated raw data
- A description of any restrictions on data availability

We collated epidemiological data from publicly available data sources (i.e., complete line list of all cases from websites of Centre for Health Protection Hong Kong: https://www.coronavirus.gov.hk/eng/index.html and https://data.gov.hk/en-data/dataset/hk-dh-chpsebcddr-novel-infectious-agent). All the epidemiological information that we used is available in the main text or the supplementary materials. The aggregate data of passenger number by card types (i.e. child, student, adult and elder) were provided in the supplementary information. Other data, including the aggregate data of passenger number by public transportation means and aggregate data of transactions by retail categories, were provided by Octopus Cards Limited (Octopus). We have obtained consent from Octopus to share the aggregate data of transport transactions between 1 January and 31 May 2020. Our agreement with Octopus prohibits us from further sharing data with third parties but interested parties can contact Octopus to make the same data request. Data and codes used in the manuscript are available in: https://github.com/kathyleung/

Octopus_mobility_model.

Field-specific reporting

Please select the one below that is the best fit for your research. If you are not sure, read the appropriate sections before making your selection.

[x] Life sciences [ ] Behavioural & social sciences [ ] Ecological, evolutionary & environmental sciences

For a reference copy of the document with all sections, see nature.com/documents/nr-reporting-summary-flat.pdf

Life sciences study design

All studies must disclose on these points even when the disclosure is negative.

Sample size	The analysis was based on the complete line list of reported COVID-19 cases in Hong Kong.<br>https://www.chp.gov.hk/files/pdf/local_situation_covid19_en.pdf<br>No sample size calculation was performed.
Data exclusions	No data were excluded.
Replication	Replication was performed for all analyses. And replication can be performed with data and codes provided in the GitHub repository: https://github.com/kathyleung/Octopus_mobility_model.
Randomization	Observational study, no randomization
Blinding	Observational study, no blinding

Reporting for specific materials, systems and methods

We require information from authors about some types of materials, experimental systems and methods used in many studies. Here, indicate whether each material, system or method listed is relevant to your study. If you are not sure if a list item applies to your research, read the appropriate section before selecting a response.

Materials & experimental systems

n/a	Involved in the study	
[x]	[ ]	Antibodies
[x]	[ ]	Eukaryotic cell lines
[x]	[ ]	Palaeontology and archaeology
[x]	[ ]	Animals and other organisms
[ ]	[x]	Human research participants
[x]	[ ]	Clinical data
[x]	[ ]	Dual use research of concern

Methods

n/a	Involved in the study	
[x]	[ ]	ChIP-seq
[x]	[ ]	Flow cytometry
[x]	[ ]	MRI-based neuroimaging

Human research participants

Policy information about studies involving human research participants

Population characteristics	The analysis was based on the complete line list of reported COVID-19 cases in Hong Kong.<br>https://www.chp.gov.hk/files/pdf/local_situation_covid19_en.pdf<br>All cases from Hong Kong were included in the analyses.
Recruitment	All cases reported to the Centre for Health Protection of Hong Kong were included in the analyses. The data are publicly available. No participant recruitment was performed.
Ethics oversight	Waived.

Note that full information on the approval of the study protocol must also be provided in the manuscript.
